# Wayside Bearing Fault Diagnosis Based on a Data-Driven Doppler Effect Eliminator and Transient Model Analysis

**DOI:** 10.3390/s140508096

**Published:** 2014-05-05

**Authors:** Fang Liu, Changqing Shen, Qingbo He, Ao Zhang, Yongbin Liu, Fanrang Kong

**Affiliations:** 1 Department of Precision Machinery and Precision Instrumentation, University of Science and Technology of China, Hefei 230026, China; E-Mails: liufang1@mail.ustc.edu.cn (F.L.); shenchangqing@mail.ustc.edu.cn (C.S.); zhangao@mail.ustc.edu.cn (A.Z.); 2 College of Electrical Engineering and Automation, Anhui University, Hefei 230093, China; E-Mail: lyb@ustc.edu.cn

**Keywords:** fault diagnosis, locomotive bearing, wayside monitoring, Doppler effect, transient model

## Abstract

A fault diagnosis strategy based on the wayside acoustic monitoring technique is investigated for locomotive bearing fault diagnosis. Inspired by the transient modeling analysis method based on correlation filtering analysis, a so-called Parametric-Mother-Doppler-Wavelet (PMDW) is constructed with six parameters, including a center characteristic frequency and five kinematic model parameters. A Doppler effect eliminator containing a PMDW generator, a correlation filtering analysis module, and a signal resampler is invented to eliminate the Doppler effect embedded in the acoustic signal of the recorded bearing. Through the Doppler effect eliminator, the five kinematic model parameters can be identified based on the signal itself. Then, the signal resampler is applied to eliminate the Doppler effect using the identified parameters. With the ability to detect early bearing faults, the transient model analysis method is employed to detect localized bearing faults after the embedded Doppler effect is eliminated. The effectiveness of the proposed fault diagnosis strategy is verified via simulation studies and applications to diagnose locomotive roller bearing defects.

## 1. Introduction

Bearing defects are the dominant type of fault in railway vehicles, which leads to serious accidents and significant costs for the rail transport industry [1]. Approximately 50 bearing-related derailments occur in the United States each year [2]. Thus, accurately and automatically detecting and diagnosing the existence and severity of these faults in the bearings are significant [3]. Wayside acoustic defective bearing detection techniques are based on the assumption that diagnostically relevant information is stored in the acoustic signal generated by the bearings of a passing vehicle. With the signal processing techniques, fault characteristic information can be extracted from the acoustic signals. A successful example is the wayside acoustic defective bearing detector (ADBD) system developed in the 1980s [4]. Compared with other systems, the ADBD system entails lower costs and can detect bearing defects earlier in the failure process or before overheating occurs, thereby allowing scheduled bearing maintenance [5].

However, the effectiveness of the wayside acoustic monitoring-based technique is decreased when vehicles pass by at high speeds. One of the problems caused by the high relative movement is the Doppler effect, as it can lead to obvious frequency shifts, frequency band expansion, and amplitude modulation for the recorded acoustic signal, which reduces the diagnostic performance [6].

Aside from the wayside acoustic monitoring system, the Doppler effect also widely exists in the signal from a moving acoustic source. For example, in the areas of underwater acoustic communication and acoustical holography for moving vehicles, the Doppler effect contained in the recorded acoustic signal is also a barrier that could significantly diminish the effectiveness of signal processing. Stojanovic *et al.* [7] and Johnson *et al.* [8] proposed a Doppler compensation method jointly based on phase synchronization and channel equalization. Yang and Wang [9] established the time-space relation among the measurement field, radiating field, and acoustic holography field; they also proposed a method based on the nonlinear mapping function between the sound source and the measured signal, in which the Doppler effect was eliminated. However, Doppler reduction methods for the wayside acoustic defective bearing detection method are rarely reported in current papers.

Dybała [10] proposed a disturbance-oriented dynamic signal resampling method based on the Hilbert Transform to eliminate the Doppler effect for wayside monitoring systems. A time-domain interpolation resampling (TIR) method is proposed by Liu [11] to remove the Doppler effect embedded in the acoustic signal. Shen [12] constructed a Doppler transient model that combines the Doppler transient model and parameter identification based on the Laplace wavelet and a spectrum correlation assessment to detect the locomotive bearing fault. However, in the above two methods, the source is assumed to move at a uniform velocity, and the sound speed is set at a constant value. In practice, the moving speed of the railway vehicle is unstable, and parameters such as the temperature and atmospheric pressure of the medium in which the wave propagates would definitely affect the value of the sound speed. These parameters could also have a significant effect on the results of the methods. What's more, all of the geometric parameters of the kinematic model are assumed to be known in advance in the proposed method.

In this paper, a fault diagnosis strategy is invented for locomotive bearing fault diagnosis based on the wayside acoustical monitoring technique. Through the proposed strategy, the Doppler effect embedded in the recorded bearing acoustic signal can be eliminated by a Doppler effect eliminator. In the Doppler effect eliminator, a so-called Parametric-Mother-Doppler-Wave (PMDW) is constructed based on the kinematic model parameters. The kinematic model parameters, including the moving speed and sound speed of the railway vehicle, as well as geometric parameters of the model are then identified via correlation filtering analysis. All of the parameters can be identified based on the signal itself. A time domain signal resampler is invented and employed to eliminate the Doppler effect using the identified kinematic model parameters. After the embedded Doppler effect is eliminated, the transient model parameters of the Doppler-free signal are identified to detect the localized bearing faults.

The rest of this paper is organized as follows: Section 2 introduces the proposed locomotive bearing fault diagnosis strategy. The construction of the PMDW, kinematic model parameter identification based on correlation filtering analysis, Doppler effect elimination based on the resampling method, and fault feature extraction based on transient model analysis are all introduced in this section. A simulation case study is provided in Section 3. An experimental verification test using defective locomotive roller bearings with outer race defect and inner race defect is discussed in Section 4. Finally, Section 5 presents the concluding remarks.

## 2. Proposed Wayside Bearing Fault Diagnosis Strategy Based on a Data-Driven Doppler Effect Eliminator and Transient Model Analysis

**Figure 1.**
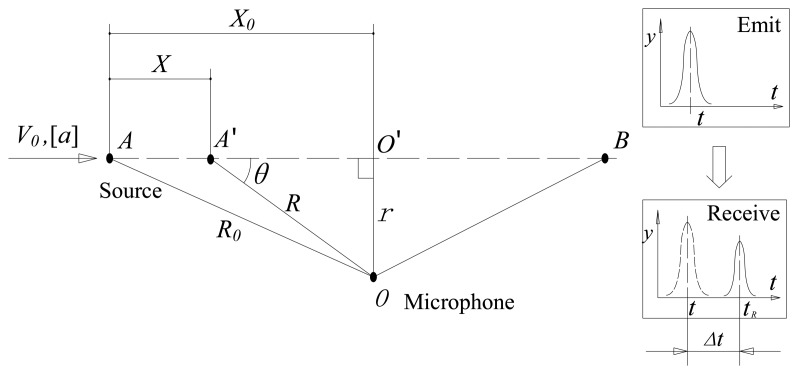
Basic kinematic model of the wayside acoustic bearing monitoring system.

In the wayside acoustic bearing monitoring system, microphones are fixed by the wayside to record the acoustic signals emitted by the bearings of a passing vehicle. The basic kinematic model (Figure 1) involving a single moving acoustic source and a microphone is considered in this study. The source is moving along a straight line, and the microphone is placed perpendicular to the trail of the moving source. When the vehicle passes by, the microphone receives the spherically attenuated signals with the Doppler effect from the bearing source.

Given the high relative speed between the railway vehicle and the microphone, the recorded signal is distorted by the Doppler effect, which causes the signal frequency to shift and the frequency band to expand. This condition is a barrier to further analysis, especially for the methods based on frequency domain analysis.

**Figure 2.**
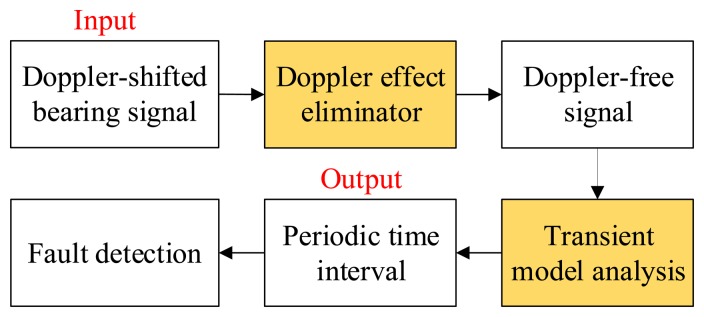
Flowchart of the proposed wayside bearing fault diagnosis strategy.

Two steps are implemented for the proposed strategy to detect the localized faults of the bearing of the fast moving railway vehicle. In the first step, the embedded Doppler effect is eliminated. The frequential structure disturbance is eliminated, and the amplitude is demodulated. In the next step, the transient model parameters of the Doppler-free signal are identified to detect the localized bearing faults. A flowchart of the proposed strategy that includes two signal processing modules is presented in Figure 2. In the following subsections, the two signal processing modules are discussed in detail.

### 2.1. Doppler Effect Eliminator

As shown in Figure 1, we assume that during the source point movement from *A* to *B*, a length of the acoustic Doppler-shifted signal, *X_dop_*(*t*), is recorded by the microphone. The kinematic model (Figure 1) can be defined by the following kinematic model parameter set:

(1) $$\gamma_{k}=\left[c,r,X_{0},V_{0},\left[a\right]\right]$$

where *c* denotes the sound speed in the air, *r* is the distance from the microphone to the trail of the moving source point, *X_0_* stands for the distance between starting point *A* and point *O′*, and point *O′* stands for the point where the source point is closest to the microphone during the pass-by movement. *V_0_* stands for the initial velocity. [*a*] is a vector containing *m* elements that stand for the 1 to *m*-order derivative of the source moving speed *V*, which is equal to the following:

(2) $$\left[a\right]=\left[\frac{dV}{dt},\frac{d^{2}V}{dt^{2}},\ldots ,\frac{d^{m}V}{dt^{m}}\right]$$

When the different structures of the passing vehicles are considered, the parameter *r* is non-constant. The speed of sound propagation *c* is unstable because of the various atmospheric environments. The parameters *X*_0_, *V_0_* and [*a*] are also difficult to measure accurately.

**Figure 3.**
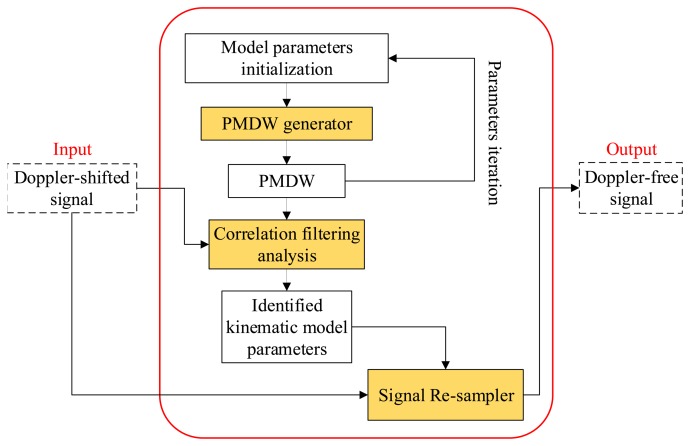
Flowchart of the Doppler effect eliminator.

A data-driven Doppler effect eliminator is invented in this paper. Through this eliminator, all of the kinematic model parameters can be identified based on the recorded signal itself. A flowchart of the eliminator is shown in Figure 3. First, the kinematic model parameter set, *γ_k_*, is identified by correlation filtering analysis between the input Doppler-shifted signal and the investigated PMDW generated by the PMDW generator. After the kinematic model parameters are identified, the Doppler-shifted signal is resampled by a signal resampler.

In the following subsection, the correlation filtering analysis method and the two signal processing modules (*i.e.*, PMDW generator and signal resampler) are discussed in detail.

#### 2.1.1. Parametric Mother-Doppler-Wave (PMDW) Generator

The construction of the PMDW is introduced in this section. As illustrated in Figure 1, we assume that the source point keeps emitting a pure harmonic sound wave during the movement from *A* to *B*. Let the harmonic sound wave be written as *S_e_* = *sin*(*2πf_c_t_e_*), *0* < *t_e_* < *T*. This wave can be sampled as a discrete amplitude sequence as Equation (3) at a sampling frequency of *f_s_*. Thus:

(3) $$S_{e}\left(n\right)=[\begin{array}{cccc} \text{sin}\left(2\pi f_{c}t_{e}\left(1\right)\right), & \text{sin}\left(2\pi f_{c}t_{e}\left(2\right)\right) & \ldots & \text{sin}\left(2\pi f_{c}t_{e}\left(N\right)\right) \end{array}]$$

that can be called “emit-amplitude-vector” and with the time sequence:

(4) $$t_{e}\left(n\right)=[\begin{array}{cccc} 0, & 1/\text{fs} & \ldots & \left(\text{N}-1\right)/\text{fs} \end{array}]$$

that can be called “emit-time-vector”. *N* equals the length of the signal. An amplitude weight of *S_e_*(*i*) is emitted from the source point at a certain emit time *t_e_*(*i*) and this amplitude weight arrives at the microphone at time *t_r_*(*i*) where the *t_r_* can be called “receive-time-vector”. Thus, we can determine that *t_r_* is equal to the following:

(5) $$t_{r}\left(n\right)=t_{e}\left(n\right)+R\left(n\right)/c$$

where *c* denotes sound speed, and *R*(*n*) stands for the distance between the source point to the microphone during the pass-by movement, which can be calculated by the following equation:

(6) $$R\left(n\right)=\sqrt{{\left[X_{0}-X\left(n\right)\right]}^{2}+r^{2}}$$

where the *X*(*n*) denotes the displacement of the source point to the initial point *A* and it can be calculated as shown in Appendix A.1.

According to the Morse acoustic theory [13], the received amplitude weight can be described by the following equation:

(7) $$S_{r}=\frac{r}{R{\left(1-M\cos\theta \right)}^{2}}\times S_{e}$$

where *θ* stands for the deflection angle of the source moving between the direction of source velocity and the microphone direction, and *M* = *V/c* stands for the mach number of the source point velocity. Thus, the “receive-amplitude-vector” can be calculated by the following equation:

(8) $$S_{r}\left(n\right)=\frac{r}{R\left(n\right)\times {\left[1-\text{V}\left(n\right)/c\times \cos\theta (n)\right]}^{2}}\times S_{e}\left(n\right)$$

where *V*(*n*) denotes the velocity of the source point equal to Appendix A.2. The cos*θ*(*n*) can be calculated by the following:

(9) cosθ(n)=X(n)/R(n)

**Figure 4.**
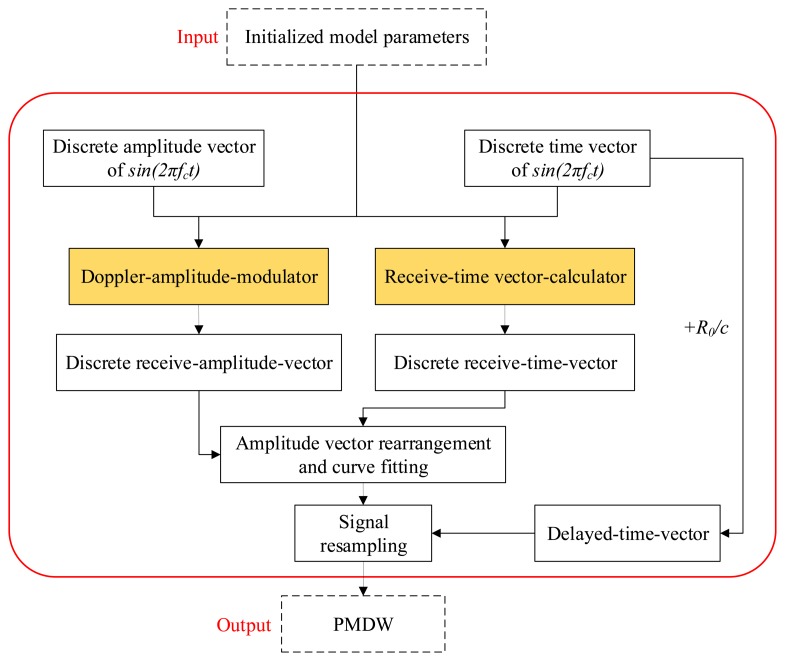
Procedure of the construction of the PMDW.

**Figure 5.**
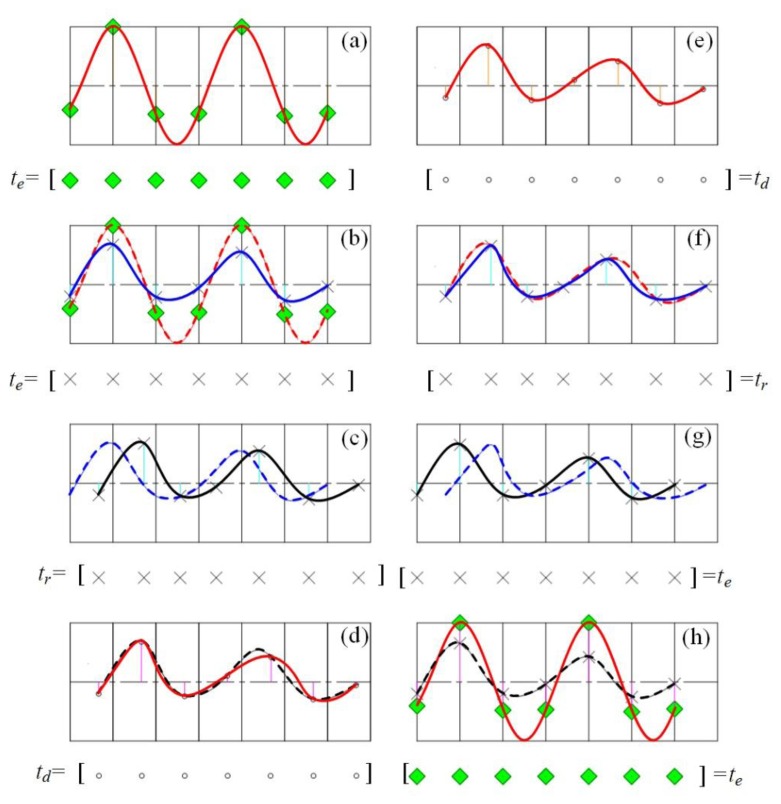
Illustration of the procedure of the construction of a PMDW and procedure of the resampler.

The detailed procedure of the PMDW construction is presented in Figure 4. A corresponding example is shown in Figure 5a–d to illustrate the procedure more clearly. The additional parameter, *f_c_*, stands for the center frequency, and the PMDW model parameter set is as follows:

(10) $$\gamma_{\Psi }=\left[f_{c},c,r,X_{0},V_{0},\left[a\right]\right]$$

These parameters belong to the subsets *S_fc_*, *S_c_*, *S_r_*, *S_X0_*, *S_V0_*, and *S_a_*, which are shown as follows:

(11) $$\begin{array}{l} S_{fc},S_{c},S_{r},S_{X0},S_{V0}\subset R^{+},\\ S_{a}\subset R \end{array}$$

Given an initialized model parameter set of γ*_Ψ_* = [*f_c_*, *c*, *r*, *X_0_*, *V_0_*, [*a*]], a PMDW can be constructed through the following steps:

(1) *Discrete sine wave generation*. The center frequency of the sine wave is *f_c_*. The discrete wave is an amplitude vector, *S_e_*, (Equation (3)) with a time vector, *t_e_*, (Equation (4)). An example is illustrated in Figure 5a.
(2) *Amplitude modulation*. Through the “Doppler-amplitude-modulator” implemented by Equation (8), a receive-amplitude-vector, *S_r_*, is obtained. The procedure is illustrated in Figure 5b.
(3) *Amplitude vector rearrangement and curve fitting*. *S_r_* obtained in Step 2 is rearranged by matching with the non-linear receive-time-vector, *t_r_*, which is calculated through the receive-time-calculator implemented by Equation (5). A new fitting curve, *χ^p^*, can then be obtained with *t_r_* and *S_r_*. The procedure is illustrated in Figure 5c.
(4) *Signal resampling*. The amplitude vector of a PMDW is then obtained by resampling the curve, *χ^p^*, obtained in Step 3 with the delayed-time-vector, *t_d_*, which is equal to *t_e_* + *R_0_/c*. *R*_0_ represents the distance between the starting point *A* and the microphone.

**Figure 6.**
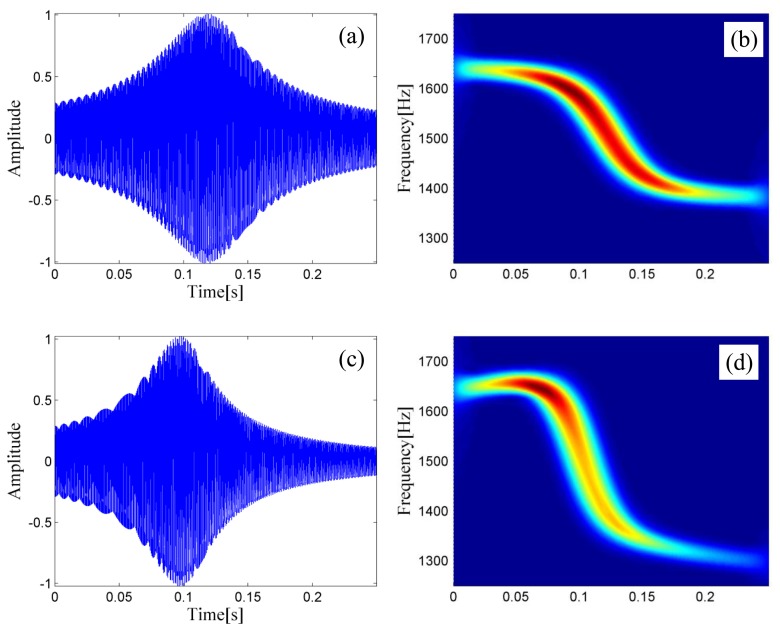
Two PMDWs generated by the invented PMDW generator. (**a**) The waveform of the PMDW generated with the model parameters set of [1,500, 340, 1, 4, 30, [0]] and (**b**) its STFT spectrum; (**c**) The waveform of the PMDW generated with the model parameters set of [1500, 340, 1, 4, 30, [100]] and (**d**) its STFT spectrum.

Two PMDWs are generated through the preceding steps. The PMDW shown in Figure 6a with its STFT spectrum in Figure 6b is generated with the following model parameter set: [1500, 340, 1, 4, 30, [0]]. The PMDW shown in Figure 6c with its STFT spectrum in Figure 6d is generated with the following parameter set: [1500, 340, 1, 4, 30, [100]].

#### 2.1.2. Improved Correlation Filtering Analysis

Correlation filtering analysis aims to measure the strength and direction of the linear relationship between two signals by calculating the Pearson's correlation coefficient between them [14]. S.B. Wang *et al.* [15] and D. Wang, *et al.* [16] employed the correlation filtering analysis method to identify the transient model parameters that were then used as rotating machine fault detection features.

In the current paper, the kinematic model parameter set described by Equation (1) is identified by an improved correlation filtering analysis between the constructed PMDWs and the recorded Doppler-shifted signal. The PMDW is first transformed into a discrete analytic wavelet through the method investigated by Marple [17]. The Pearson's correlation coefficient between the recorded Doppler-shifted signal and the real part, as well as the imaginary part of the analytic wavelet, are then calculated. Finally, the square root of the two obtained coefficients is employed as a criterion of the inherent linear relationship between the two signals. Through this improved correlation filtering analysis method, the consideration of the initial phase of the two signals could be ignored, as the imaginary part of the analytic wavelet is a 90-degree phase shift signal of the PMDW.

Given an input Doppler-shifted signal, *X_dop_*(*n*), and a parametric wavelet, *Ψ*(*n*), we can implement the improved correlation filtering analysis through the following procedure:

(1) The discrete-time analytic signal of the parametric wavelet is obtained:
  (12) $$A*\left[\Psi_{\gamma }\left(n\right)\right]=\Psi_{\gamma }\left(n\right)+H\left[\Psi_{\gamma }\left(n\right)\right]$$
(2) The Pearson's correlation coefficients are calculated as follows:
  (13) $$\rho_{X_{\text{dop}}\left(n\right),\text{R}\left(n\right)}=\frac{\sum_{i=1}^{N}\left(X_{\text{dop}}\left(i\right)-{\bar{X}}_{\text{dop}}\right)\left(\text{R}\left(i\right)-\bar{\text{R}}\right)}{\sqrt{\sum_{i=1}^{N}{\left(X_{\text{dop}}\left(i\right)-{\bar{X}}_{\text{dop}}\right)}^{2}}\sqrt{\sum_{i=1}^{N}{\left(\text{R}\left(i\right)-\bar{\text{R}}\right)}^{2}}}$$
  (14) $$\rho_{X_{\text{dop}}\left(n\right),I\left(n\right)}=\frac{\sum_{i=1}^{N}\left(X_{\text{dop}}\left(i\right)-{\bar{X}}_{\text{dop}}\right)\left(I\left(i\right)-\bar{\text{I}}\right)}{\sqrt{\sum_{i=1}^{N}{\left(X_{\text{dop}}\left(i\right)-{\bar{X}}_{\text{dop}}\right)}^{2}}\sqrt{\sum_{i=1}^{N}{\left(I\left(i\right)-\bar{\text{I}}\right)}^{2}}}$$
  where *R*(*n*) is the real part of A* [*Ψ_γ_*(*n*)], and *I*(*n*) is the imaginary part of A* [*Ψ_γ_*(*n*)], *X̄_dop_* stands for the mean of *X_dop_*, *R̄* and *Ī* stand for the mean of *R* and *I* respectively.
(3) The criterion of the inherent linear relationship between *x*(*n*) and *Ψ*(*n*) is as follows:
  (15) $$\rho_{X_{\text{dop}}\left(n\right),\Psi_{\gamma }\left(\text{n}\right)}=\sqrt{{\rho^{2}}_{X_{\text{dop}}\left(n\right),\text{R}\left(\text{n}\right)}+{\rho^{2}}_{X_{\text{dop}}\left(n\right),\text{I}\left(\text{n}\right)}}$$

#### 2.1.3. Signal Re-Sampler

After the kinematic model parameters are identified, the Doppler-shifted signal and the identified kinematic model parameters are inputted into the signal resampler, through which the embedded Doppler effect can be clearly eliminated.

If the identified kinematics model parameters are as follows:

(16) $${\gamma^{\text{opt}}}_{k}=\left[c^{\text{opt}},r^{\text{opt}},{X_{0}}^{\text{opt}},{V_{0}}^{\text{opt}},\left[a^{\text{opt}}\right]\right]$$

and if the input Doppler-shifted signal is *S_r_^dop^*(*n*) with a length of *N^dop^* points, then the variables similar to those mentioned in Section 2.1.1 can be calculated as follows:

Emit-time-vector:

(17) $${t_{e}}^{\text{dop}}\left(n\right)=[\begin{array}{cccc} 0, & 1/\text{fs} & \ldots & \left({\text{N}}^{\text{dop}}-1\right)/\text{fs} \end{array}]$$

where *N^dop^* equals to the length of the input Doppler-shifted signal.

Receive-time-vector:

(18) $${t_{r}}^{\text{dop}}\left(n\right)={t_{e}}^{\text{dop}}\left(n\right)+R^{\text{dop}}\left(n\right)/c$$

where *R^dop^* (*n*) equals:

(19) $$R^{\text{dop}}\left(n\right)=\sqrt{{\left[{X_{0}}^{\text{opt}}-X^{\text{dop}}\left(n\right)\right]}^{2}+r^{opt^{2}}}$$

where *X^dop^*(*n*) equals to Appendix A.3.

According to Equation (7), the “emit-amplitude-vector” can be calculated by the following equation:

(20) $${S_{e}}^{\text{dop}}\left(n\right)={S_{r}}^{\text{dop}}\left(n\right)\times {\left[\frac{r^{\text{opt}}}{R^{\text{dop}}\left(n\right)\times {\left[1-{\text{V}}^{\text{dop}}\left(n\right)/{\text{c}}^{\text{opt}}\times \cos\theta^{\text{dop}}(\text{n})\right]}^{2}}\right]}^{-1}$$

where *V^dop^*(*n*) equals to Appendix A.4.

and cos*θ^dop^* (*n*) equals:

(21) $$\cos\theta^{\text{dop}}\left(n\right)=X^{\text{dop}}\left(n\right)/R^{\text{dop}}\left(n\right)$$

**Figure 7.**
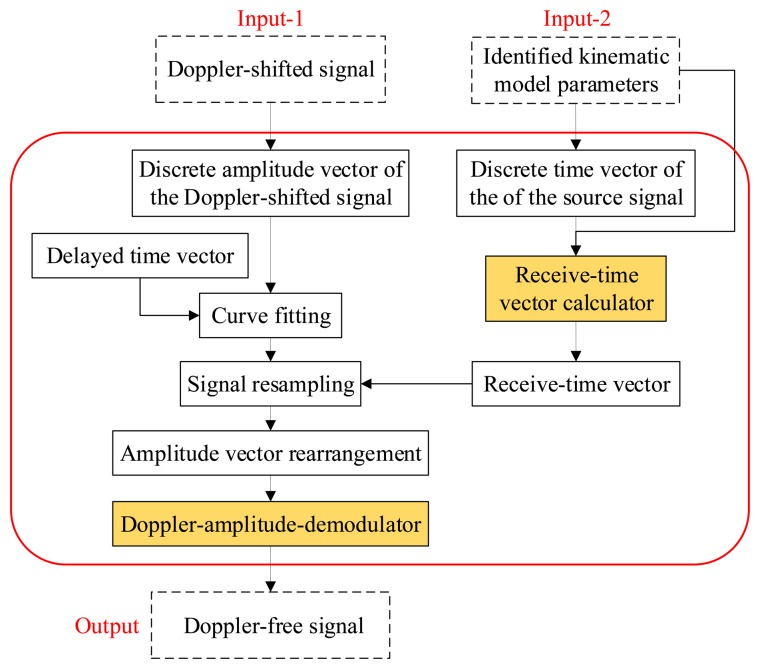
Procedure of the signal resampler.

The detailed procedure of the signal resampler is shown in Figure 7. A corresponding example is shown in Figure 5e–h to illustrate the procedure more clearly.

Given a certain identified kinematic model parameter set of *γ_k_* = [*c^opt^*, *r^opt^*, *X_0_^opt^*, *V_0_^opt^*, [*a^opt^*]], the procedure of the resampler can be described as follows:

(1) *Curve fitting*. The amplitude vector of the input Doppler-shifted signal is matched with the delayed-time-vector, *t_d_^dop^*, which equals [*R_0_^dop^/c^opt^*, *R_0_^dop^/c^opt^* + *1/f_s_*, …, *R_0_^dop^/c^opt^* + *(N^dop^-1)/f_s_*]. A fitting curve, *χ^d^*, can then be obtained by fitting the amplitude of the Doppler-shifted signal with *t_d_^dop^*. An example is illustrated in Figure 5e.
(2) *Signal resampling*. The receive-amplitude-vector, *S_r_^dop^*(*n*), is then obtained by resampling the curve, *χ^d^*, obtained in Step 1 with the receive-time-vector, *t_r_^dop^*, shown in Equation (18). This procedure is illustrated in Figure 5f.
(3) *Amplitude vector rearrangement*. The receive-amplitude-vector, *S_r_*(*n*), obtained in Step 2 is rearranged by matching with the linear emit-time-vector, *t_e_^dop^*, shown in Equation (17). This procedure is illustrated in Figure 5g.
(4) *Amplitude demodulation*. The Doppler-free signal can be obtained by demodulating the rearranged amplitude vector in Step 2 through the “Doppler-amplitude-demodulator” implemented by Equation (20). This procedure is illustrated in Figure 5h.

### 2.2. Transient Model Analysis

The Doppler effect is eliminated through the aforementioned data-driven Doppler effect eliminator. Conventional fault feature extraction methods can be employed to analyze the Doppler-free signal, extract features, and then make a maintenance decision. In the past decades, numerous methods have been proposed to extract features for bearing signals, such as time-domain analysis [18], frequency-domain analysis [19], time-frequency-domain analysis [20–23], envelope spectrum [24,25], wavelet transform [26–29], empirical mode decomposition [30–32], and manifold learning [33].

**Figure 8.**
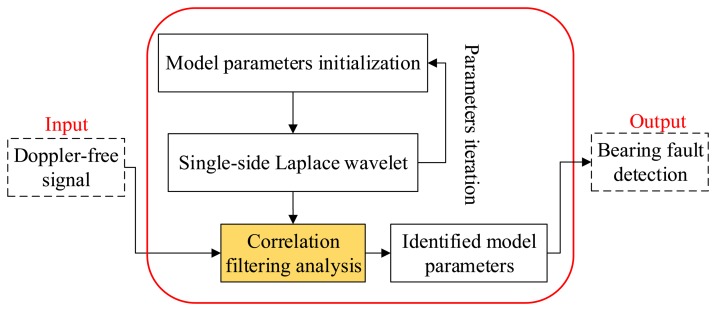
Procedure of the transient model analysis method.

A method of transient modeling by wavelet and parameter identification based on correlation filtering is first introduced and applied on bearing fault diagnosis by Wang *et al.* [15]. Whenever a bearing suffers a localized fault, the transients with a potential cyclic characteristic are generated by the rollers striking the localized fault. This phenomenon is an early bearing fault feature. The extraction of the transients is therefore beneficial to identify an early bearing fault [16]. The method is employed in this section to detect the localized defect of the locomotive bearing. A flowchart of this method is presented in Figure 8. It follows the steps of transient model construction, parameter identification through the correlation filtering analysis method, and bearing fault type identification through the recognized impact periods. Each step is discussed in detail in the following subsections.

#### 2.2.1. Transient Model Construction Based on the Laplace Wavelet

The rolling element bearing typically consists of an inner race, an outer race, a number of rolling elements, and a cage. Once a localized fault is formed on the surface of the inner or outer race, a transient with an exponential decay is generated by the roller striking the localized fault.

The Laplace wavelet, a single-sided damped exponential function formulated as the impulse response of a single mode system, is highly similar to the waveform feature commonly encountered in bearing fault signal detection tasks.

The results reported in reference [15] show that the real part of the complex Laplace wavelet was the most sensitive wavelet to the transients generated by the localized bearing faults. The formula of the real part of the Laplace wavelet is as follows:

(22) $$\psi_{\text{Laplace}}(f,\zeta ,\tau ,t)=\left\{ \begin{array}{l} e^{-\zeta /\sqrt{1-\zeta^{2}}2\pi f\times (t-\tau )}\cos(2\pi f\times (t-\tau )),\tau \leq t\leq \tau +W\\ 0,\text{else} \end{array}\right.$$

where *W* is the temporal range, *f* is the discrete frequency, *ζ* is the discrete damping coefficient, and *τ* is the delay time. These parameters belong to subsets *F*, *Z*, and *T_d_*, which are shown as follows:

(23) $$\begin{array}{c} F\subset R^{+}\\ Z\subset (R^{+}\cap [0,1))\\ T\subset R^{+} \end{array}$$

The speed variation has been removed during the procedure of Doppler effect elimination, however, the train of transients is not a strict periodic phenomenon when considering the “jitter” [34] during the operation. When considering this ‘jitter’, a different model should be used [35] and the traditional methods need to be modified [34]. The studies in this paper are based on the hypothesis that the train of transients is periodic after the speed variation has been removed. Then a periodic multi-transient model based on the Laplace wavelet is therefore constructed to simulate the waveform characteristics by introducing parameter *T*, as follows:

(24) $$\chi \left(t\right)=\sum_{m}\psi_{\text{Laplace}}(f,\zeta ,\tau ,t-mT)$$

**Figure 9.**
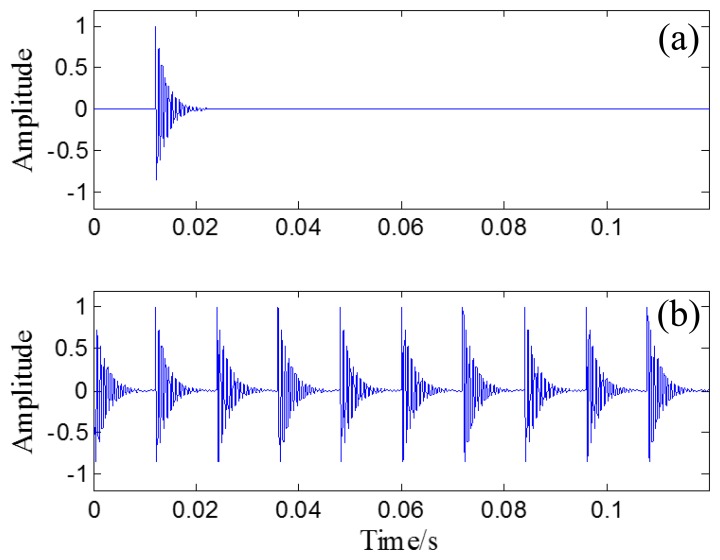
(**a**) Single Laplace wavelet transient model (**b**) periodic Laplace wavelet transient model.

Figure 9 illustrates the single and periodic Laplace wavelet transient models, respectively.

#### 2.2.2. Locomotive Bearing Fault Detection by Transient Model Parameters Identification

If the surface of the outer race of the bearing suffers a single defect based on the bearing geometries and rotation speed, *f_r_*, the ball passing frequency over the outer race defect (BPFO) can be calculated as follows:

(25) $$\text{BPFO}=\frac{1}{2}(1-\frac{d}{D_{m}}\cos\alpha )f_{n}Z$$

where *d* and *D_m_* represent the diameter of the rolling elements and the pitch diameter, respectively. *α* denotes the contact angle, *f_n_* denotes the rotational frequency, and *Z* is the number of rolling elements.

Similarly, if a single defect occurs on the surface of the inner race of the bearing, the ball pass frequency over the inner race defect (BPFI) can be obtained by the following:

(26) $$\text{BPFI}=\frac{1}{2}(1+\frac{d}{D_{m}}\cos\alpha )f_{n}Z$$

Every time the rolling element passes through the defect, periodic impulses are created with time interval, *Δt*, as follows:

(27) $$\Delta t=\frac{1}{\text{BPFO}}$$

or:

(28) $$\Delta t=\frac{1}{\text{BPFI}}$$

After the Doppler effect embedded in the acoustic signal of the bearing is eliminated. The time interval can be identified through the improved correlation filtering analysis introduced in Section 2.1.2 between the periodic multi-transient model shown in Equation (24) and the Doppler-free signal of the bearing. The identified impact period in the transient model is the related bearing fault impact interval. The fault type can be determined by referring to the calculated theoretical fault-related impact intervals.

## 3. Simulation Case Study

In this section, a simulated Doppler-shifted bearing signal is analyzed to verify the effectiveness of the investigated diagnosis strategy. The source signal of the bearing without the Doppler effect can be described as follows:

(29) $$X\left(t\right)=e^{-\zeta_{0}/\sqrt{1-{\zeta_{0}}^{2}}2\pi f_{0}\mathrm{mod}(\text{t},1/40)}\cos(2\pi f_{0}t)+n\left(t\right),t=0:1/50000:9999/50000$$

where the damping ratio, *ζ*_0_, and frequency, *f*_0_, are set at 0.05 and 1000 Hz, respectively. The impact interval embedded in the simulated signal is 0.025 *s*. The number of data points is 10,000, and the sampling frequency is 50 kHz.

**Figure 10.**
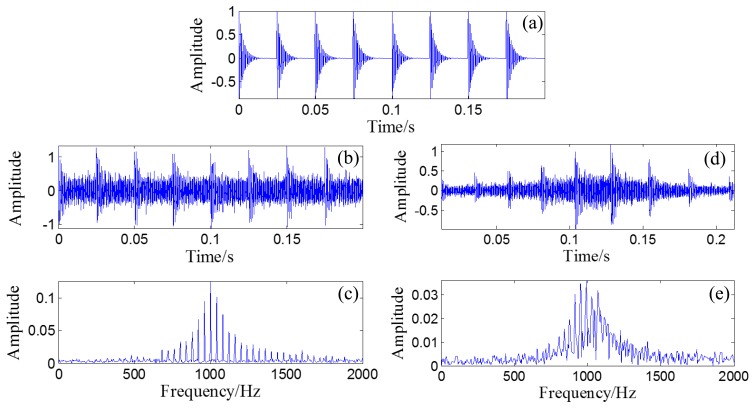
(**a**) Waveform of the simulated bearing signal without noise; (**b**) Waveform of the simulated bearing signal with noise and (**c**) its FFT spectrum; (**d**) Waveform of the simulated Doppler-shifted bearing signal with noise and (**e**) its FFT spectrum.

A randomly distributed noise, *n*(*t*), is added to the simulated signal. The waveform of the simulated signal without noise is illustrated in Figure 10a. The polluted signal is shown in Figure 10b with its FFT spectrum in Figure 10c.

Through the PMDW generator introduced in Section 2.1.1, a sine wave can be embedded with the Doppler effect. If the sine wave is replaced with the aforementioned simulated signal, a Doppler-shifted bearing signal can be obtained with the same procedure and with the following kinematic parameters: *c* = 340 m/s, *r* = 1 m, *X_0_* = 4 m, *V_0_* = 30 m/s, [*a*] = [*a_1_*] = 40 m/s^2^. The wave form and FFT spectrum of the simulated Doppler-shifted bearing signal are illustrated in Figure 10d,e, respectively.

The proposed diagnosis strategy is then applied to the Doppler-shifted bearing signal. The Doppler-shifted bearing signal is first inputted to the Doppler effect eliminator introduced in Section 2.1 to eliminate the embedded Doppler effect. Through the eliminator, the kinematic parameters are first identified through the correlation filtering analysis introduced in Section 2.1.2 between the input Doppler-shifted signal and the PMDWs constructed with parameter subsets described by Equation (11).

The selection of the parameter subsets is crucial. On the one hand, the larger interval range and the smaller step of the parameter subset obtain a more accurate result. On the other hand, the larger interval range and the smaller step of the parameter subset cost excessive computation and decrease the efficiency of the method. When both accuracy and efficiency are considered, the subset, *S_fc_*, is determined as [800:10:1200] by inspecting the FFT spectrum of the Doppler-shifted signal. The sound speed is set as *S_c_* = [320:1:360]. The parameter sets *S_r_*, *S_X0_*, and *S_V0_* are set as [0.5:0.1:1.5], [2:0.1:6] and [20:0.1:40], respectively. Only the first-order derivative of the moving speed, *a_1_*, is considered, and *S_a_* is set as [35:0.5:45].

**Figure 11.**
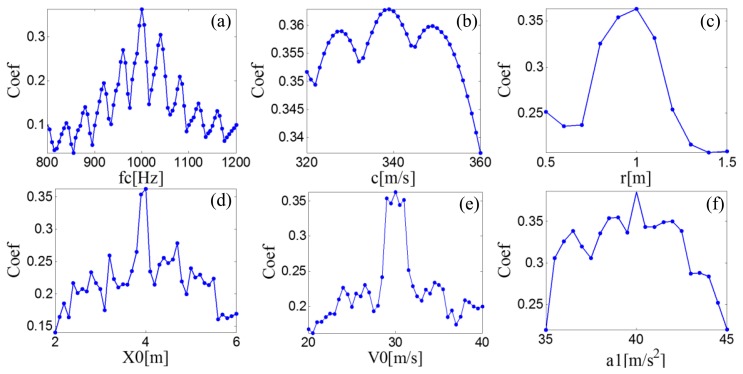
Maximal correlation coefficients for the different elements from a specified PMDW model parameter subset for the simulated signal.

A searching grid of the model parameters is constructed based on the aforementioned six parameter subsets. Once a group of parameters is determined, the parameters are inputted to the PMDW generator introduced in Section 2.1.1 to generate a PMDW. The correlation filtering analysis introduced in Section 2.1.2 is then performed between the PMDW and the simulated Doppler-shifted signal. Figure 11 shows the maximal correlation coefficients for the different elements from a specified model parameter subset. When the parameter set of the PMDW is determined as Table 1, the maximal correlation coefficient between the PMDW and the simulated Doppler-shifted signal can be obtained.

**Figure 12.**
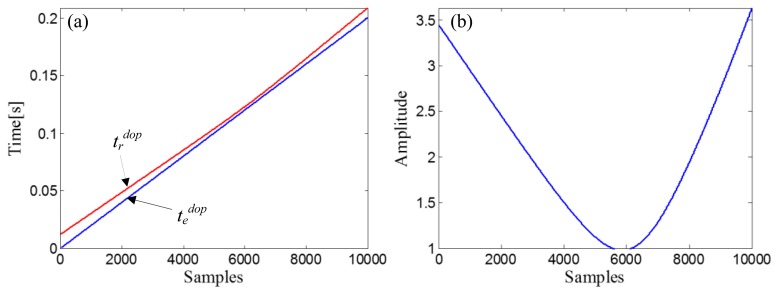
(**a**) Receive-time-vector (red curve) and the emit-time-vector (blue curve) in the simulation case; (**b**) Curve of the demodulation weights in the simulation case.

The identified kinematic model parameters and the Doppler-shifted bearing signal are then inputted to the signal resampler introduced in Section 2.1.3 to eliminate the embedded Doppler effect. First, the amplitude vector of the input Doppler-shifted signal is matched with the delayed-time-vector, *t_d_^dop^* = [*R_0_^dop^/c^opt^*, *R_0_^dop^/c^opt^* + *1/f_s_*,…, *R_0_^dop^/c^opt^* + *(N^dop^*−*1)/f_s_*], where $R_{0}=\sqrt{r^{opt^{2}}+{X_{0}}^{opt^{2}}}=\sqrt{1^{2}+4^{2}}=4.1231$, and a fitting curve, *χ^d^*, can be obtained by fitting the amplitude vector of the input Doppler-shifted signal with *t_d_^dop^*. Through the receive-time-calculator described by Equation (18), the receive-time-vector, *t_r_^dop^*, can be calculated with the emit-time-vector, *t_e_^dop^* = [0,1/*f_s_*,…, (10000−1)/*f_s_*]. The two time vectors are shown in Figure 12a. After resampling the fitting curve, *χ^d^*, by *t_r_^dop^*, we obtain the receive-amplitude-vector, *S_r_^dop^*(*n*). Finally, the obtained *S_r_^dop^*(*n*) is rearranged by matching with *t_e_^dop^* and then demodulated by Equation (20). The curve of the demodulation weights is indicated in Figure 12b.

**Figure 13.**
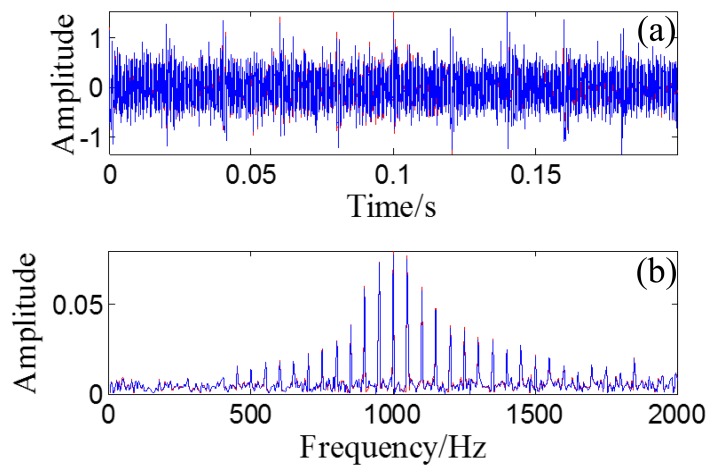
Comparison between the obtained Doppler-free signal and the original simulated bearing signal (**a**) wave form of the Doppler-free signal (red) and the original simulated bearing signal (blue); (**b**) FFT spectrum of the Doppler-free signal (red) and FFT spectrum of the original simulated bearing signal (blue).

The wave form of the obtained Doppler-free signal is plotted as an overlay on the original bearing signal, shown in Figure 13a, with their FFT spectrums in Figure 13b. The correlation coefficient between the obtained Doppler-free signal and the original bearing signal is 0.9695, which indicates that the Doppler effect is effectively eliminated.

**Figure 14.**
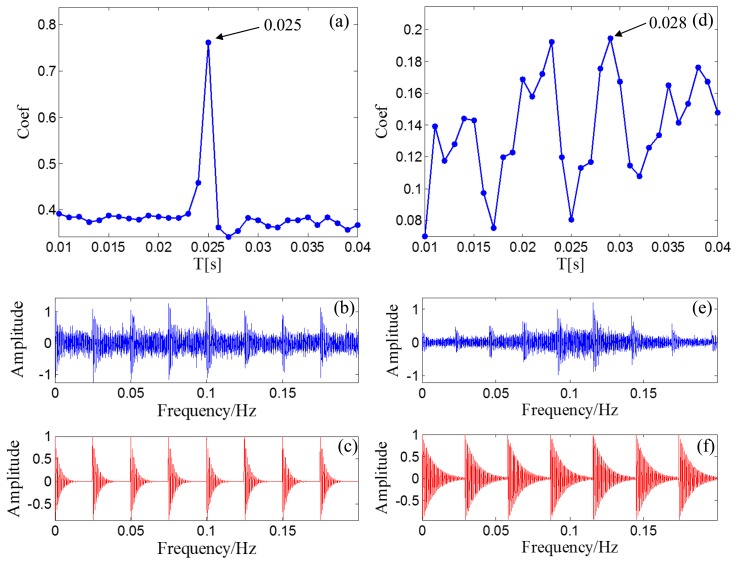
Results for simulated Doppler-shifted bearing signal using the transient model analysis method. (**a**) maximal correlation coefficients for different elements from set *T* after Doppler effect elimination; (**b**) wave form of the Doppler-free signal; (**c**) the optimal periodical transient model for the Doppler-free signal; (**d**) maximal correlation coefficients for different elements from set *T* before Doppler effect elimination; (**e**) wave form of the Doppler-shifted signal; (**f**) the optimal periodical transient model for the Doppler-shifted signal.

After the Doppler effect is eliminated, the transient model analysis method introduced in Section 2.2 is applied to the Doppler-free signal to detect the defect. The transient model is constructed according to Equation (24). Its parameters require optimization from the sets *T*, *F*, and *Z*. The range of *T*, *F*, and *Z* are set as [500/50,000:1/50,000:2000/50,000], [800:10:1200], and { [0.005:0.001:0.03] ∪ [0.04:0.01:0.1] ∪ [0.2:0.1:0.9]}, respectively. The subset of *Z* is non-uniform to provide higher resolution at lower damping ratio values so that the efficiency of the method can be retained. The grid of the model parameters is constructed according to *F* and *Z* for each element from set *T*. When a group of parameters is determined, the transient model is constructed according to the procedures introduced in Section 2.2.1. The correlation filtering analysis introduced in Section 2.1.2 is then performed between the transient model and the input Doppler-free bearing signal. Figure 14a shows the maximal correlation coefficients for the different elements from set *T*. The optimal parameters *f_c_* = 1000 and ζ = 0.05 when *T* is set at 0.025 s.

As a comparison, the simulated signal before the Doppler effect elimination is also analyzed via the transient model analysis method with the same model parameter subsets. The maximal correlation coefficients for the different elements from set *T* are shown in Figure 14b. The maximal correlation coefficient is obtained when the impact period is 0.028 s, which is an incorrect value. Comparison of the transmit model analysis results between the signals before and after Doppler effect elimination is shown in Table 2. Thus, performing the introduced Doppler effect elimination method before the conventional transient model analysis is necessary.

## 4. Application in Locomotive Bearings Fault Diagnosis

### 4.1. Experimental Setup and Data Acquisition

Two separate experiments were implemented to achieve the Doppler-shifted acoustic signals from defective locomotive roller bearings and verify the effectiveness of the proposed method. The obtained Doppler-shifted bearing acoustic signals are then analyzed by the proposed diagnosis strategy.

**Figure 15.**
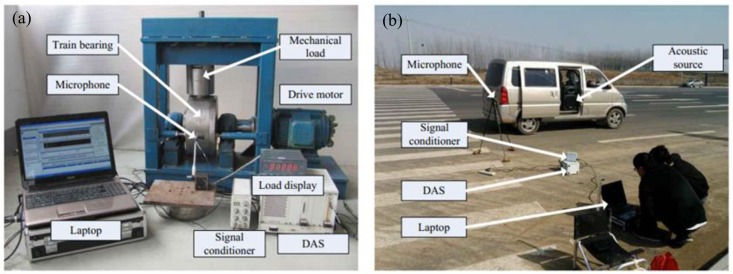
Experimental setup for signal acquisition with Doppler effect (**a**) test bench of the first experiment and (**b**) scene of the second experiment.

In the first experiment, the acoustic signal of a locomotive roller bearing with a single localized defect was acquired by the test bench in Figure 15a. The test bench consists of a drive motor, two supporting pillow blocks (mounted with normal bearings), a testing bearing loaded on the outer race, and a mechanical radial loader. A 4944-A type of microphone (B&K Company, Nærum, Denmark) was mounted adjacent to the outer race of the tested bearing for acoustic signal measurement. The advanced data acquisition system (DAS) provided by National Instruments (Austin, TX, US) was used to record the data. The specifications of the testing bearing are listed in Table 3. The bearings were tested under the radial load of 3 t, the rotation speed of the motor was set at 1430 r/min, and the sampling frequency was set at 50 kHz.

The second experiment can be referred to the model illustrated in Figure 1. As shown in Figure 15b, the experiment consists of a moving vehicle (mounted with the acoustic source), a same microphone, and a same DAS as those in the first experiment. When the car passes by the microphone, a Doppler-shifted signal of the testing bearing can be received and recorded. In this experiment, the car was passing by at an accelerated speed. The sampling frequency was also set at 50 kHz.

**Figure 16.**
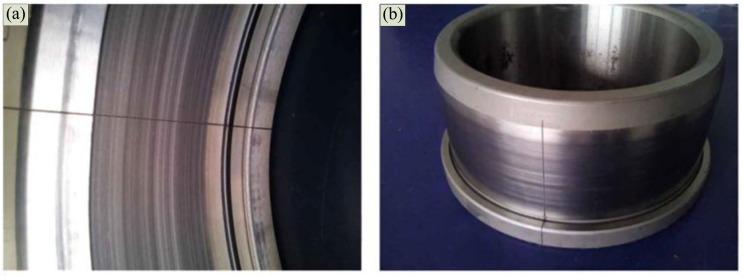
Artificial defects on the components of the bearing (**a**) defect on the outer race and (**b**) defect on the inner race.

A single artificial crack with 0.18 mm width was set by the wire-electrode cutting machine on the outer and inner race, as shown in Figure 16. The Doppler-shifted bearing signals were then acquired by the aforementioned experimental steps. In the subsequent sections, the Doppler-shifted signals with the out-race defect (size: 0.18 mm) and inner-race defect (size: 0.18 mm) are analyzed.

### 4.2. Results

**Figure 17.**
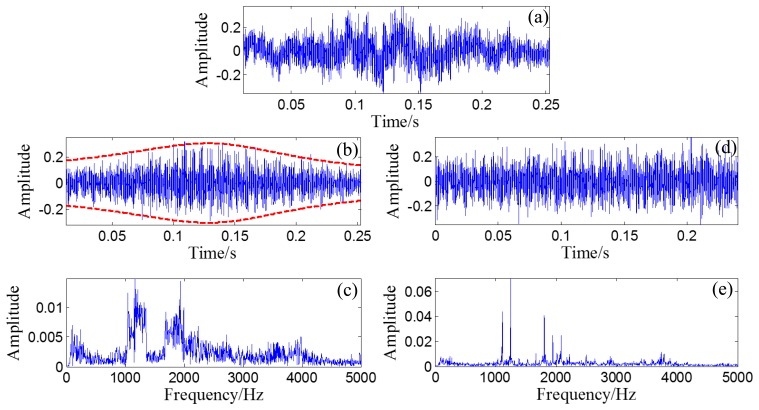
(**a**) Waveform of the Doppler-shifted signal with the out-race defect; (**b**) waveform of the filtered Doppler-shifted signal with the out-race defect and (**c**) its FFT spectrum; (**d**) waveform of the obtained Doppler-free signal with the out-race defect and (**e**) its FFT spectrum.

The wave form of the Doppler-shifted bearing signal with the out-race defect is shown in Figure 17a with a length of 12,000 points. As the original recorded signal is highly distorted by the low-frequency component and our study shows that the low-frequency component will reduce the effectiveness of the following correlation analysis. So a fourth-order Butterworth band-pass filter is employed to reduce this distortion. And our study also shows that if the band of the filter is too narrow, for example just around the structural resonance band, the effectiveness of the following correlation analysis will be reduced as the waveform of the signal will be damaged by the filter. So, the filter band is set as 50 Hz to 5 kHz. The wave form of the filtered signal is presented in Figure 17b with its FFT spectrum in Figure 17c.

The proposed diagnosis strategy is then applied to the filtered Doppler-shifted bearing signal. The signal is first inputted to the Doppler effect eliminator introduced in Section 2.1 to identify the kinematic model parameters and eliminate the embedded Doppler effect. During the kinematic parameter identification procedure, the subset *S_fc_* is determined as [800:10:1600] by inspecting the FFT spectrum in Figure 17c. The sound speed is set as *S_c_* = [320:1:360], determined by the value of 343.2 m/s that stands for the sound speed in standard air atmosphere.

The value of sound speed *c* is determined by the properties of the medium of air. In the Earth's atmosphere, the chief factor affecting the sound speed is the temperature. In practice, it can be estimated through the value calculated by the following practical formula:

(30) $$c_{\text{air}}=331.3+0.606T$$

The parameter sets *S_r_* and *S_X0_* are set as [1.5:0.1:2.5] and [2:0.1:6], respectively, based on a previous coarse manual measurement. As the momentum of the car is large and the time duration of the pass-by movement is extremely short, only the first-order derivative of the moving speed, *a_1_*, is considered. *S_V0_* and *S_a_* is set as [20:0.1:40] and [0:1:10], respectively, by considering the acceleration performance of the testing car. In parctice, the range of initial speed can also be estimated by the length of the recorded signal and the dimension of the vehicle.

**Figure 18.**
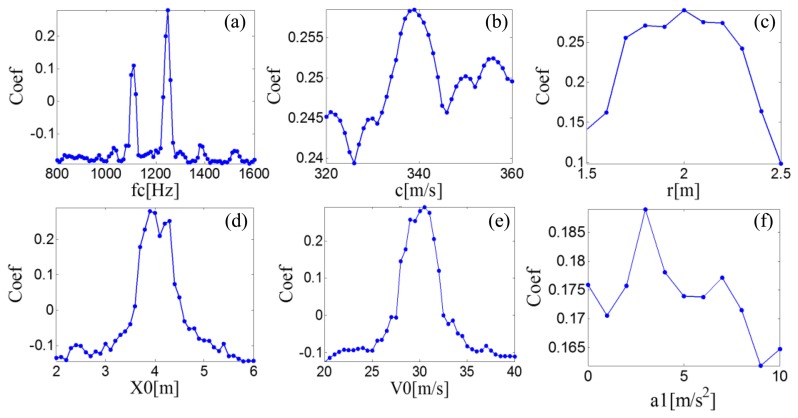
Maximal correlation coefficients for the different elements from a specified PMDW model parameter subset for the Doppler-shifted signal with the out-race defect.

PMDWs are then generated with different parameters from the previously determined parameter subsets by the PMDW generator introduced in Section 2.1.1 to generate a PMDW. The correlation filtering analysis introduced in Section 2.1.2 is then performed between the generated PMDW and the Doppler-shifted signal. Figure 18 shows the maximal correlation coefficients for the different elements from a specified parameter subset. When the parameter set of the PMDW is determined as those in Table 4, the maximal correlation coefficient between the PMDW and the Doppler-shifted signal can be obtained.

**Figure 19.**
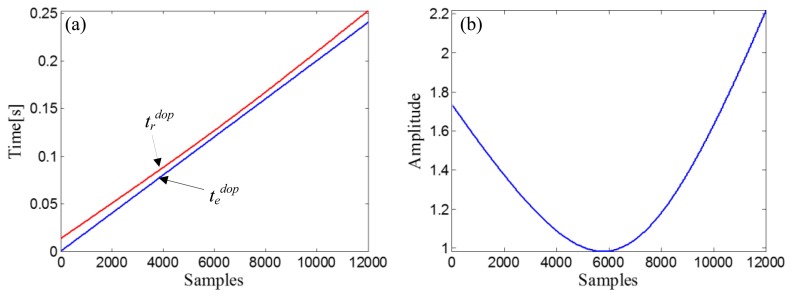
(**a**) Receive-time-vector (red curve) and the time vector of the source signal (blue curve) and (**b**) Curve of the demodulation weights, in the experimental case study of the bearing signal with the out-race defect.

The signal resampler introduced in Section 2.1.3 is then employed to eliminate the embedded Doppler effect with the identified kinematic parameters in Table 4. The amplitude vector of the input Doppler-shifted signal is matched with the delayed-time-vector, *t_d_^dop^*, which equals [*R_0_^dop^/c^opt^*, *R_0_^dop^/c^opt^* + *1/f_s_*,…, *R_0_^dop^/c^opt^* + *(N^dop^*−*1)/f_s_*], where *R_0_^dop^* equals $\sqrt{r^{opt^{2}}+{X_{0}}^{opt^{2}}}=\sqrt{2^{2}+3.9^{2}}=4.3829$, and a fitting curve, *χ^d^*, can be obtained by fitting the amplitude vector of the input Doppler-shifted signal with *t_d_^dop^*. The receive-time-vector, *t_r_^dop^*, can be calculated from the emit-time-vector, *t_e_^dop^* = [0,1/*f_s_*,…, (12000−1)/*f_s_*], through the receive-time-calculator described by Equation (18). The two time vectors are shown in Figure 19a. The next step is resampling the fitting curve, *χ^d^*, by *t_r_^dop^* to obtain the receive-amplitude-vector, *S_r_^dop^*(*n*). After the amplitude vector rearrangement by matching with *t_e_^dop^* and the amplitude demodulation procedure implemented by Equation (20), the curve of the demodulation weights can be obtained (Figure 19b).

The wave form of the obtained Doppler-free signal is shown in Figure 17d, with its FFT spectrum in Figure 17e. Compared with the shape of the FFT spectrum in Figure 17c, the problems of frequency shift and frequency band expansion are clearly solved. When Figure 17d is compared with Figure 17b, the amplitude is also clearly demodulated.

The transient model analysis method introduced in Section 2.2 is then applied to detect the characteristic interval of the Doppler-free fault signal. A periodical transient model with parameters adjustable using Equation (24) is constructed from the sets *T* = [50/50000:1/50000:600/50000], *F* = [800:5:2400], and *Z* = { [0.005:0.001:0.03] ∪ [0.04:0.01:0.1] ∪ [0.2:0.1:0.9]}. When a group of parameters is determined, the transient model is constructed according to the procedures introduced in Section 2.2.1. The correlation filtering analysis introduced in Section 2.1.2 is then performed between the transient model and the input Doppler-free bearing signal.

**Figure 20.**
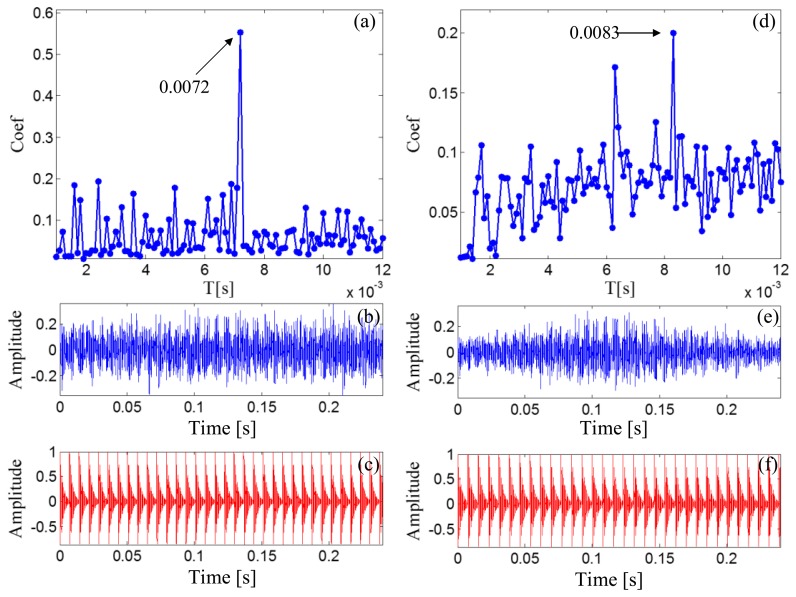
Results for Doppler-shifted bearing signal with the out-race defect using the transient model analysis method (**a**) maximal correlation coefficients for different elements from set *T* after Doppler effect elimination; (**b**) wave form of the Doppler-free signal; (**c**) the optimal periodical transient model for the Doppler-free signal; (**d**) maximal correlation coefficients for different elements from set *T* before Doppler effect elimination; (**e**) wave form of the Doppler-shifted signal; (**f**) the optimal periodical transient model for the Doppler-shifted signal.

The outer race characteristic frequency is 138.74 Hz as calculated by Equation (25), and the periodical impact interval is 0.0072 s as calculated by Equation (27). Figure 20a reports the maximal correlation coefficients for the different elements from set *T*. The optimal impact period is 0.0072 s, which is equal to the real bearing fault-related impact interval. The optimal transient model is shown in Figure 20c.

The signal before the Doppler effect elimination is also analyzed via transient model analysis method with the same model parameter subsets. The maximal correlation coefficients for the different elements from set *T* are shown in Figure 20d. The maximal correlation coefficient is obtained when the impact period is 0.0083 s, which is incorrect.

**Figure 21.**
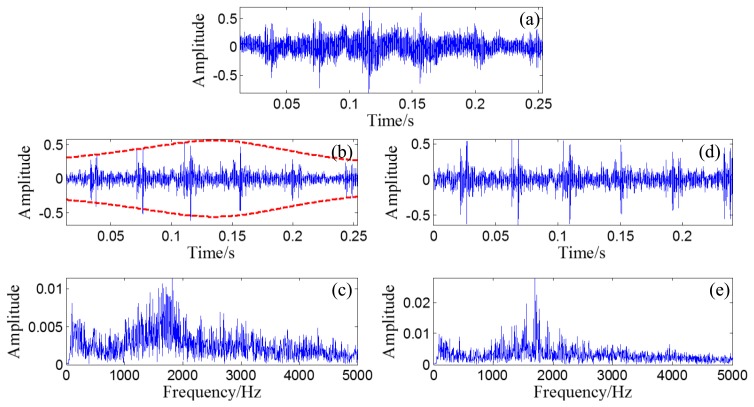
(**a**) Waveform of the Doppler-shifted signal with the inner-race defect; (**b**) waveform of the filtered Doppler-shifted signal with the inner-race defect and (**c**) its FFT spectrum; (**d**) waveform of the Doppler-free signal with the inner-race defect and (**e**) its FFT spectrum.

The obtained Doppler-shifted bearing signal (Figure 21a) with an inner-race defect is also analyzed to further confirm the effectiveness of the proposed fault diagnosis strategy. Thus, the signal was also first pre-processed with a four-order Butterworth band-pass filter (band: 50 Hz to 5 kHz). The wave form of the filtered signal is shown in Figure 21b, with its FFT spectrum in Figure 21c.

**Figure 22.**
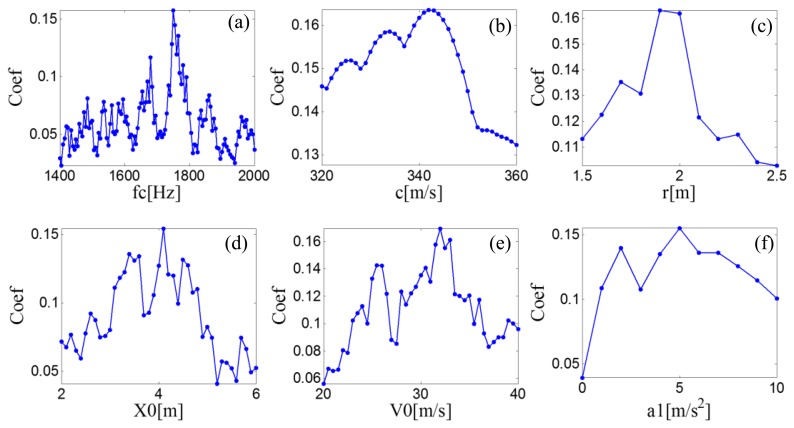
Maximal correlation coefficients for the different elements from a specified PMDW model parameter subset for the Doppler-shifted signal with the inner-race defect.

The proposed Doppler effect eliminator is first applied to identify the kinematic model parameters and eliminate the embedded Doppler effect. The subset *S_fc_* is determined as [1400:10:2000] by inspecting the FFT spectrum in Figure 21c. The parameter sets *S_c_*, *S_r_*, *S_X0_*, *S_V0_*, and *S_a_*, are set as [320:1:360], [1.5:0.1:2.5], [2:0.1:6], [20:0.1:40], and [0:1:10], respectively. PMDWs are then generated with different parameters from the determined parameter subsets by the PMDW generator introduced in Section 2.1.1 to generate a PMDW. The correlation filtering analysis introduced in Section 2.1.2 is then performed between the generated PMDWs and the simulated Doppler-shifted signal. Figure 22 shows the maximal correlation coefficients for the different elements from a specified parameter subset. When the parameter set of the PMDW is determined as those in Table 5, the maximal correlation coefficient between the PMDW and the simulated Doppler-shifted signal can be obtained.

**Figure 23.**
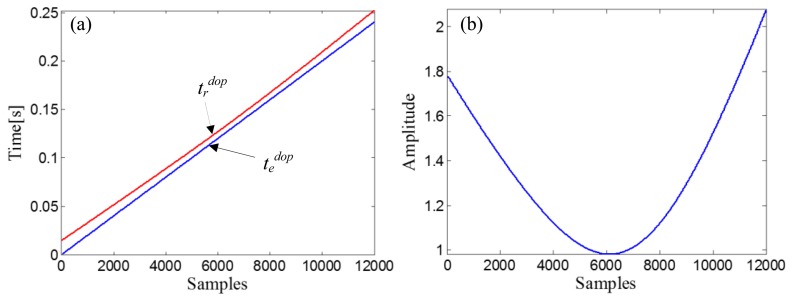
(**a**) Receive-time-vector (red curve) and the time vector of the source signal (blue curve) and (**b**) Curve of the demodulation weights, in the experimental case study of the bearing signal with the inner-race defect.

The embedded Doppler effect is then eliminated through the signal resampler introduced in Section 2.1.3. First, by matching the amplitude vector of the input Doppler-shifted signal with the delayed-time-vector, *t_d_^dop^*, which equals [*R_0_^dop^/c^opt^*, *R_0_^dop^/c^opt^* + *1/f_s_*,…, *R_0_^dop^/c^opt^* + *(N^dop^*−*1)/f_s_*], where *R_0_^dop^* equals $\sqrt{r^{opt^{2}}+{X_{0}}^{opt^{2}}}=\sqrt{1.9^{2}+4.1^{2}}=4.5188$, a fitting curve, *χ^d^*, can be obtained. Second, the receive-time-vector, *t_r_^dop^*, is calculated from the emit-time-vector, *t_e_^dop^* = [0,1/*f_s_*,…, (12000−1)/*f_s_*], through the receive-time-calculator described by Equation (18). The two time vectors are shown in Figure 23a. After resampling the fitting curve, *χ^d^*, by *t_r_^dop^*, the receive-amplitude-vector, *S_r_^dop^*(*n*), is obtained. Finally, the amplitude vector is rearranged by matching with *t_e_^dop^* and performing the amplitude demodulation procedure through Equation (20). The curve of the demodulation weights is shown in Figure 23b.

The wave form of the obtained Doppler-free signal is illustrated in Figure 21d with its FFT spectrum in Figure 21e. Compared with the shape of the FFT spectrum in Figure 21c and the wave form in Figure 21b, the problems of frequency shift, frequency band expansion, and amplitude modulation are clearly solved.

The transient model analysis method introduced in Section 2.2 is then applied to detect the characteristic interval embedded in the Doppler-free fault signal. A periodical transient model with parameters adjustable using Equation (24) is constructed from the sets *T* = [50/50000:1/50000:600/50000], *F* = [1200:5:2200], and *Z* = { [0.005:0.001:0.03] ∪ [0.04:0.01:0.1] ∪ [0.2:0.1:0.9]}. Figure 22a shows the maximal correlation coefficients for the different elements from set *T*.

**Figure 24.**
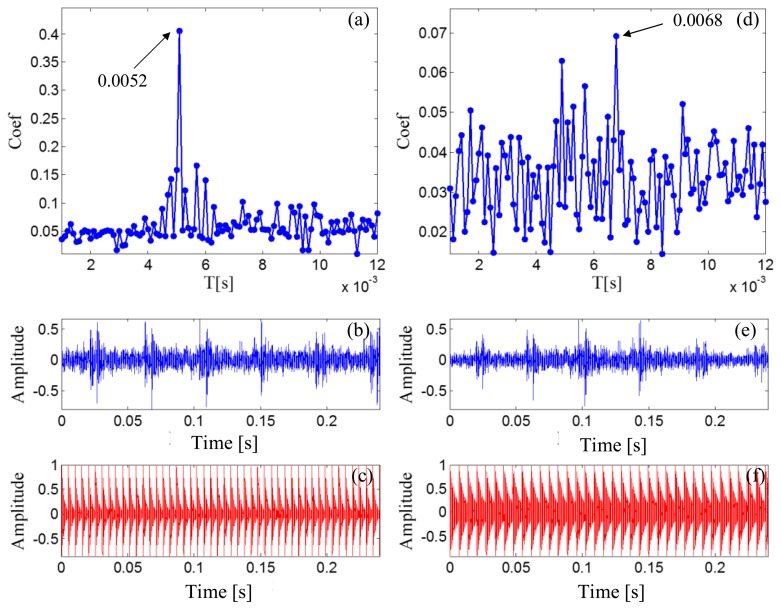
Results for Doppler-shifted bearing signal with the inner-race defect using the transient model analysis method (**a**) maximal correlation coefficients for different elements from set *T* after Doppler effect elimination; (**b**) wave form of the Doppler-free signal; (**c**) the optimal periodical transient model for the Doppler-free signal; (**d**) maximal correlation coefficients for different elements from set *T* before Doppler effect elimination; (**e**) wave form of the Doppler-shifted signal; (**f**) the optimal periodical transient model for the Doppler-shifted signal.

The periodical impact interval is 0.0052 s, calculated by Equations (29) and (31). Figure 24a shows that the optimal impact period equals the real bearing fault-related impact interval. The optimal transient model is shown in Figure 24c.

The Doppler-shifted signal is then directly analyzed by the transient model analysis method with the same model parameter subsets. The maximal correlation coefficients for the different elements from set *T* are shown in Figure 24d. The maximal correlation coefficient is obtained when the impact period is 0.0068 s, which is an incorrect value.

## 5. Conclusions

In this paper, a fault diagnosis strategy based on the wayside acoustic monitoring technique is invented for locomotive bearing fault diagnosis. A parametric wavelet called PMDW is introduced and employed to identify the kinematic model parameters based on correlation analysis. A time domain signal resampler is introduced and employed to eliminate the embedded Doppler effect in the recorded bearing acoustic signal. The transient model analysis method is also employed to detect the localized bearing faults after the Doppler effect is eliminated. One of the best benefits of the proposed strategy is that all the kinematic model parameters, including the sound speed and the moving speed of the vehicle, as well as the geometric parameters of the model, can be identified based on the recorded signal itself. Thus, the proposed strategy overcomes the difficulties of kinematic model parameter measurement and is adjustable to different types of passing vehicles. Besides, the embedded Doppler effect can be eliminated through the proposed strategy, paving the way for the conventional invented signal processing methods and feature extraction methods. The performance of the proposed strategy has been evaluated by both simulated and practical Doppler-shifted bearing signals carrying fault information. Given the merits revealed in this study, the proposed fault diagnosis strategy can be widely used in wayside health monitoring systems, particularly in situations when vehicles pass by at high moving speeds and kinematic model parameters are difficult to estimate. The proposed data-driven Doppler effect eliminator is also hopeful to be used in other areas such as acoustic communication techniques and sound field holography for moving vehicles.

## Figures and Tables

**Table 1.** Kinematic model parameters subset and the optimal kinematic model parameters of the simulated signal.

**Items**	***f****_c_* **[Hz]**	***c* [m/s]**	***r* [m]**	***x****_0_* **[m]**	***v****_0_* **[m/s]**	***a****_0_* **[m/s^2^]**
Range	[800,1200]	[320,360]	[0.5,1.5]	[2,6]	[20,40]	[35,45]
Step	10	1	0.1	0.1	0.1	0.5
Optimal value	1000	340	1	4	30	40
True value	1000	340	1	4	30	40
Error	0%	0%	0%	0%	0%	0%

**Table 2.** Comparison of the transmit model analysis results between the signals before and after Doppler effect elimination.

**Items**	***Coef***	***f****_res_*	***ζ***	***T***
Before Doppler effect elimination	0.195	1060	0.019	0.028
After Doppler effect elimination	0.780	1000	0.050	0.025

**Table 3.** Specifications of the testing bearing.

**Type**	**NJ(P)3226XI**
Diameter of the outer race	250 mm
Diameter of the inner race	130 mm
Pitch diameter (*D*)	190 mm
Diameter of the roller (*d*)	32 mm
Number of the roller (*z*)	14

**Table 4.** Kinematic model parameter subsets and the optimal kinematic model parameters of the bearing signal with the out-race defect.

**Items**	***f****_c_* **[Hz]**	***c* [m/s]**	***r* [m]**	***x****_0_* **[m]**	***v****_0_* **[Hz]**	***a****_0_* **[Hz^2^]**
Range	[800:1600]	[320:360]	[1.5:2.5]	[2:6]	[20:40]	[0:10]
Step	10	1	0.1	0.1	0.1	1
Optimal value	1250	339	2	3.9	30.5	3

**Table 5.** Kinematics model parameters subset and the optimal kinematics model parameters of the bearing signal with the inner-race defect.

**Items**	***f****_c_* **[Hz]**	***c* [m/s]**	***r* [m]**	***x****_0_* **[m]**	***v****_0_* **[m/s]**	***a****_0_* **[m/s^2^]**
Range	[1400:2000]	[320:360]	[1.5:2.5]	[2:6]	[20,40]	[0,10]
Step	10	1	0.1	0.1	0.1	1
Optimal value	1750	342	1.9	4.1	32	5

## Appendix A

### A.1. Displacement of the Source Point to the Initial Point A

$$\begin{array}{l} \begin{array}{ll} X\left(n\right) & =V_{0}\times t_{e}+{\left[\text{t}\right]}^{X}\times [\text{a}]\\ & =V_{0}\times \left[\begin{array}{c} t_{e}\left(1\right)\\ t_{e}\left(2\right)\\ \ldots \\ t_{e}\left(N\right) \end{array}\right]+{\left[\begin{array}{cccc} \frac{1}{2!}{t_{e}}^{2}\left(1\right) & \frac{1}{3!}{t_{e}}^{3}\left(1\right) & \ldots & \frac{1}{\left(\text{m}+1\right)!}{t_{e}}^{\left(m+1\right)}\left(1\right)\\ \frac{1}{2!}{t_{e}}^{2}\left(2\right) & \frac{1}{3!}{t_{e}}^{3}\left(2\right) & \ldots & \frac{1}{\left(m+1\right)!}{t_{e}}^{\left(m+1\right)}\left(2\right)\\ \ldots & \ldots & \ldots & \ldots \\ \frac{1}{2!}{t_{e}}^{2}\left(N\right) & \frac{1}{3!}{t_{e}}^{3}\left(N\right) & \ldots & \frac{1}{\left(m+1\right)!}{t_{e}}^{\left(m+1\right)}\left(N\right) \end{array}\right]}_{N\times m}\times \left[\begin{array}{c} a_{1}\\ a_{2}\\ \ldots \\ a_{\text{m}} \end{array}\right] \end{array} \end{array}$$

### A.2. Velocity of the Source Point

$$\begin{array}{l} V\left(n\right)=V_{0}+{\left[\text{t}\right]}^{V}\times [a]\\ =V_{0}+{\left[\begin{array}{cccc} t_{e}\left(1\right) & \frac{1}{2!}{t_{e}}^{2}\left(1\right) & \ldots & \frac{1}{\text{m}!}{t_{e}}^{m}\left(1\right)\\ t_{e}\left(2\right) & \frac{1}{2!}{t_{e}}^{2}\left(2\right) & \ldots & \frac{1}{\text{m}!}{t_{e}}^{m}\left(2\right)\\ \ldots & \ldots & \ldots & \ldots \\ t_{e}\left(N\right) & \frac{1}{2!}{t_{e}}^{2}\left(N\right) & \ldots & \frac{1}{\text{m}!}{t_{e}}^{m}\left(N\right) \end{array}\right]}_{N\times m}\times \left[\begin{array}{c} a_{1}\\ a_{2}\\ \ldots \\ a_{\text{m}} \end{array}\right] \end{array}$$

### A.3. Expression of X^dop^(n)

$$\begin{array}{l} X^{\text{dop}}\left(n\right)={V_{0}}^{\text{opt}}\times {t_{e}}^{\text{dop}}+{\left[\text{t}\right]}^{X^{\text{dop}}}\times [{\text{a}}^{\text{opt}}]\\ ={V_{0}}^{\text{opt}}\times \left[\begin{array}{c} {t_{e}}^{\text{dop}}\left(1\right)\\ {t_{e}}^{\text{dop}}\left(2\right)\\ \ldots \\ {t_{e}}^{\text{dop}}\left(N^{\text{dop}}\right) \end{array}\right]+\\ {\left[\begin{array}{cccc} \frac{1}{2!}{t_{e}}^{dop^{2}} & \frac{1}{3!}{t_{e}}^{\text{dop}}{\left(1\right)}^{3} & \ldots & \frac{1}{\left(m^{\text{dop}}+1\right)!}{t_{e}}^{\text{dop}}{\left(1\right)}^{m^{\text{dop}}+1}\\ \frac{1}{2!}{t_{e}}^{\text{dop}}{\left(2\right)}^{2} & \frac{1}{3!}{t_{e}}^{\text{dop}}{\left(2\right)}^{3} & \ldots & \frac{1}{\left(m^{\text{dop}}+1\right)!}{t_{e}}^{\text{dop}}{\left(2\right)}^{m^{\text{dop}}+1}\\ \ldots & \ldots & \ldots & \ldots \\ \frac{1}{2!}{t_{e}}^{\text{dop}}{\left(N^{\text{dop}}\right)}^{2} & \frac{1}{3!}{t_{e}}^{\text{dop}}{\left(N^{\text{dop}}\right)}^{3} & \ldots & \frac{1}{\left(m^{\text{dop}}+1\right)!}{t_{e}}^{\text{dop}}{\left(N^{\text{dop}}\right)}^{m^{\text{dop}}+1} \end{array}\right]}_{N^{\text{dop}}\times m^{\text{dop}}}\times \left[\begin{array}{c} {a_{1}}^{\text{opt}}\\ {a_{2}}^{\text{opt}}\\ \ldots \\ {a_{m}}^{\text{opt}} \end{array}\right] \end{array}$$

### A.4. Expression of V^dop^(n)

$$\begin{array}{l} V^{\text{dop}}\left(n\right)={V_{0}}^{\text{opt}}+{\left[\text{t}\right]}^{a^{\text{opt}}}\times [{\text{a}}^{\text{opt}}]\\ ={V_{0}}^{\text{opt}}+{\left[\begin{array}{cccc} {t_{e}}^{\text{dop}}\left(1\right) & \frac{1}{2!}{t_{e}}^{\text{dop}}{\left(1\right)}^{2} & \ldots & \frac{1}{{\text{m}}^{\text{dop}}!}{t_{e}}^{\text{dop}}{\left(1\right)}^{{\text{m}}^{\text{dop}}}\\ {t_{e}}^{\text{dop}}\left(2\right) & \frac{1}{2!}{t_{e}}^{\text{dop}}{\left(2\right)}^{2} & \ldots & \frac{1}{{\text{m}}^{\text{dop}}!}{t_{e}}^{\text{dop}}{\left(2\right)}^{{\text{m}}^{\text{dop}}}\\ \ldots & \ldots & \ldots & \ldots \\ {t_{e}}^{\text{dop}}\left(N^{\text{dop}}\right) & \frac{1}{2!}{t_{e}}^{\text{dop}}{\left(N^{\text{dop}}\right)}^{2} & \ldots & \frac{1}{{\text{m}}^{\text{dop}}!}{t_{e}}^{\text{dop}}{\left(N^{\text{dop}}\right)}^{{\text{m}}^{\text{dop}}} \end{array}\right]}_{N^{\text{dop}}\times m^{\text{dop}}}\times \left[\begin{array}{c} {a_{1}}^{\text{opt}}\\ {a_{2}}^{\text{opt}}\\ \ldots \\ {a_{m}}^{\text{opt}} \end{array}\right] \end{array}$$
